# Nuclear Factor-Erythroid 2-Related Factor 2 (Nrf2) and Mitochondrial Dynamics/Mitophagy in Neurological Diseases

**DOI:** 10.3390/antiox9070617

**Published:** 2020-07-15

**Authors:** Tae-Cheon Kang

**Affiliations:** 1 Department of Anatomy and Neurobiology, College of Medicine, Hallym University, Chuncheon 24252, Korea; tckang@hallym.ac.kr; Tel.: +82-33-248-2524; Fax: +82-33-248-2525; 2 Institute of Epilepsy Research, College of Medicine, Hallym University, Chuncheon 24252, Korea

**Keywords:** Alzheimer’s disease, cerebrovascular disease, epilepsy, Huntington’s disease, mitochondrial fission, mitochondrial fusion, Parkinson’s disease

## Abstract

Mitochondria play an essential role in bioenergetics and respiratory functions for cell viability through numerous biochemical processes. To maintain mitochondria quality control and homeostasis, mitochondrial morphologies change rapidly in response to external insults and changes in metabolic status through fusion and fission (so called mitochondrial dynamics). Furthermore, damaged mitochondria are removed via a selective autophagosomal process, referred to as mitophagy. Although mitochondria are one of the sources of reactive oxygen species (ROS), they are themselves vulnerable to oxidative stress. Thus, endogenous antioxidant defense systems play an important role in cell survival under physiological and pathological conditions. Nuclear factor-erythroid 2-related factor 2 (Nrf2) is a redox-sensitive transcription factor that maintains redox homeostasis by regulating antioxidant-response element (ARE)-dependent transcription and the expression of antioxidant defense enzymes. Although the Nrf2 system is positively associated with mitochondrial biogenesis and mitochondrial quality control, the relationship between Nrf2 signaling and mitochondrial dynamics/mitophagy has not been sufficiently addressed in the literature. This review article describes recent clinical and experimental observations on the relationship between Nrf2 and mitochondrial dynamics/mitophagy in various neurological diseases.

## 1. Introduction

Mitochondria play an essential role in bioenergetics and respiratory functions for cell viability through numerous biochemical processes, such as oxidative phosphorylation (OXPHOS), the Krebs cycle and β-oxidation of fatty acids [1,2,3]. In order to maintain cellular homeostasis, mitochondria continuously change their morphologies (referred to as mitochondrial dynamics) in response to the extra- and intracellular microenvironment. Under physiological conditions, excess nutrients induce mitochondrial fragmentation (fission) [1]. In contrast, deprivation of nutrients facilitates mitochondrial elongation (fusion) to increase OXPHOS activity and the mitochondrial content exchange [2,3]. These mitochondrial dynamics allow the adaptation of mitochondrial activity to physiological demands, as required for ATP production, Ca^2+^ homeostasis and regulation of reactive oxygen species (ROS) production [4]. Furthermore, damaged mitochondria are removed by mitophagy (a selective autophagosomal process of mitochondrial degradation) to maintain mitochondria quality control and homeostasis [5]. Thus, dysfunctions of mitochondrial dynamics/mitophagy are relevant to the pathogenesis of metabolic diseases, various cancers, and neurodegenerative diseases [6,7]. For example, dysfunctions of mitochondrial metabolism and/or imbalances of mitochondrial dynamics are involved in the pathophysiology of obesity and diabetes [8]. Type 2 diabetes is related to impaired mitochondrial fusion in skeletal muscle, which depresses OXPHOS [9,10]. Mitochondrial fragmentation decreases ATP content, which leads to reduced insulin-mediated glucose uptake and loss of glucose-stimulated insulin secretion in pancreatic β cells [11,12]. In addition, fragmented mitochondrial networks in liver and skeletal muscle result in glucose intolerance and enhanced hepatic gluconeogenesis [13,14]. Mitochondrial fission is also relevant for the highly active glycolysis in rapid proliferating cancer cells, since the limited mitochondrial oxidation in fragmented mitochondria preserves glycolytic intermediates that are used for cancer cell proliferation [8]. Mutations of genes related to mitochondrial fusion cause Charcot–Marie–Tooth disease (a peripheral neuropathy characterized by axonal degeneration and distal muscular atrophy) and autosomal dominant optic atrophy (the most prevalent form of inherited optic neuropathy in humans) [8,15,16]. Impaired mitochondrial fission also leads to a fatal neonatal disorder characterized by congenital microcephaly, optic atrophy, optic hypoplasia, lactic acidosis, and elevated serum levels of very long-chain fatty acids [17].

Since mitochondria are vulnerable to oxidative stress, endogenous antioxidant defense systems play an important role in cell survival under physiological and pathological conditions. Nuclear factor-erythroid 2-related factor 2 (Nrf2) is a redox-sensitive transcription factor that maintains redox homeostasis by regulating antioxidant-response element (ARE)-dependent transcription and the expression of antioxidant defense enzymes [18,19]. However, the relationship between Nrf2 signaling and mitochondrial dynamics/mitophagy has not been sufficiently elaborated in the literature. This review article focuses on recent experimental observations on the relationship between Nrf2 signaling and mitochondrial dynamics/mitophagy in various neurological diseases.

## 2. Nrf2 and Mitochondrial Functions

Oxidative stress by ROS triggers mitochondrial dynamics. In addition, mitochondrial fission directly facilitates an increase in mitochondrial ROS production. During electron transport, superoxide (O_2_^−^) is generated as a result of electron leak from the electron transport chain and one-electron reduction of oxygen. ROS induces mitochondrial fragmentation as a physiological event [20,21]. A cellular response to increased metabolic substrate, mitochondrial fragmentation increases accessibility of metabolic substrate to carrier proteins, and facilitates metabolic input into mitochondria. However, mitochondrial fragmentation also affects the structural and spatial organization of the respiratory chain and ATP synthase by changing electron transport, which leads to mitochondrial hyperpolarization and ROS production. Indeed, inhibition of mitochondrial fragmentation prevents ROS production [20,21].

**Figure 1:**
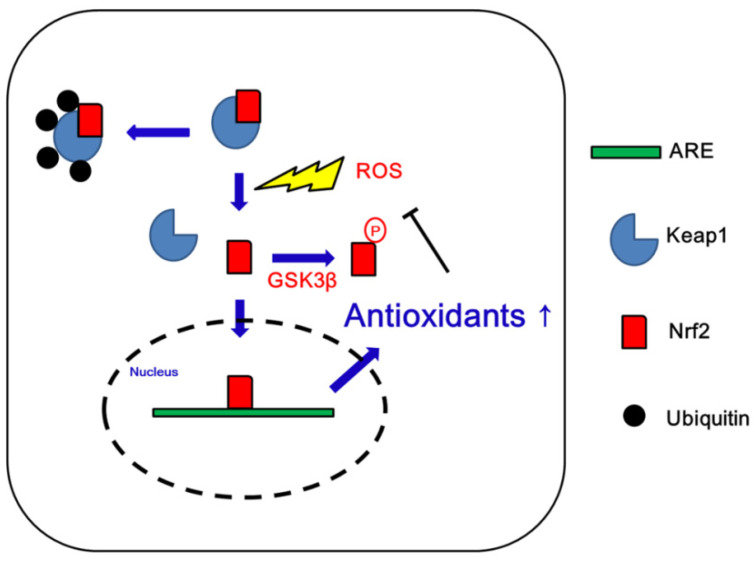
Schematic depiction of the regulation of Nrf2 in a normal cell. Abbreviations: ARE, antioxidant response element; GSK3β, glycogen synthase kinase 3β; Keap1, Kelch-like ECH-associated protein 1; Nrf2, nuclear factor-erythroid 2-related factor 2; ROS, reactive oxygen species.

Increased ROS levels activate cellular antioxidant defense mechanisms that play an important role in mitochondrial dynamics/mitophagy under physiological and pathological conditions. Nrf2 is a transcription factor that induces antioxidant and detoxifying enzymes to protect cells from oxidative stress [22,23,24]. Thus, Nrf2 contributes to a broad spectrum of cellular functions such as redox balance, cell cycle, cell death, immunity, metabolism, selective protein degradation, development, aging, and carcinogenesis. Nrf2 forms a heterodimer with small Maf proteins and binds to antioxidant response elements (AREs) in the promoter region of its target genes [22,23,24]. Under physiological conditions, Nrf2 is bound to its cytoplasmic repressor Kelch-like ECH-associated protein 1 (Keap1), which prevents Nrf2 translocation into the nucleus. Keap1 also mediates Nrf2 polyubiquitinylation and subsequent proteasomal degradation through the cullin-3 (Cul3)-based E3 ubiquitin ligase complex [22,23,24]. Under oxidative stress, oxidation of SH-groups in Keap1 inhibits its ability to target Nrf2 for degradation. In turn, Nrf2 escapes from Keap1 binding and translocates into the nucleus where it transactivates multiple ARE-bearing genes, such as glutathione S-transferases (GSTs), NAD(P)H:quinone oxidoreductase 1 (NQO1), thioredoxin, thioredoxin reductase, ROS scavengers and glutathione (GSH) synthetic enzymes [22,23,24]. Glycogen synthase kinase 3β (GSK3β) also represses Nrf2 via phosphorylation that inhibits Nrf2 translocation to the nucleus [25,26]. Thus, the Nrf2-Keap1 protein complex acts as a cellular redox sensor and maintains redox homeostasis by regulating the transcription of antioxidant genes (Figure 1).

Since Nrf2 activates the antioxidant systems in response to oxidative stress, Nrf2 signaling is one of the primary systems counteracting mitochondria-derived ROS. Indeed, Nrf2 plays a critical role in maintaining the mitochondrial GSH pool by elevating GSH biosynthesis. In addition, Nrf2 increases the level of NADPH (an important reducing molecule in the cell), which is used for the GSH reductase-mediated generation of reduced GSH and GSH peroxidase (GPx)-mediated removal of hydrogen peroxide. Nrf2 also directly regulates the expression of mitochondrial antioxidant enzymes; superoxide dismutase 2 (SOD2), peroxiredoxin 3 (Prdx3), Prdx5, GPx1, and thioredoxin reductase 2 (TrxR2). In addition, Nrf2 activation increases the expression levels of nuclear respiratory factor-1 (NRF-1, also known as α-palindromic-binding protein (α-PAL)) and mitochondrial transcription factor A (TFAM), which in turn modulate the expression of mitochondrial respiratory subunits and translational components [27,28,29,30,31,32,33].

Peroxisome proliferator-activated receptor (PPAR)-α promotes the utilization and catabolism of fatty acids by upregulating genes involved in fatty acid transport and peroxisomal/mitochondrial fatty acid β-oxidation [34]. PPAR-γ is involved in glucose metabolism and regulation of fatty acid storage, adipocyte differentiation (adipogenesis), insulin sensitivity and cell growth [35]. Both PPAR-α and PPAR-γ activate members of the PPAR-γ coactivator (PGC) family that regulate mitochondrial biogenesis [36]. PPAR agonists, such as bezafibrate, thiazolidinedione, pioglitazone and rosiglitazone, activate PPAR and PGC-1α to increase mitochondrial biogenesis and reduce mitochondrial dysfunction [37]. PGC-1α promotes mitochondrial SOD2 expression by interacting with Nrf2, which contributes to maintaining mitochondrial mass and redox homeostasis [33].

ATP synthase (complex V of the electron transport chain) produces ATP via the influx of protons back into the matrix. The mitochondrial membrane potential (MMP) is the difference in proton concentration across the inner membrane, which is indicative of the mitochondrial capacity for ATP generation [28,33]. Nrf2 also activates MMP, oxygen consumption rate, fatty acid oxidation and the TCA cycle in mitochondria [27,28,29]. Given that Nrf2 controls mitochondrial functions through mitochondrial antioxidant defense, bioenergetics and biogenesis, it is plausible that Nrf2 may also affect mitochondrial dynamics/mitophagy under physiological and pathological conditions. However, direct experimental evidence for a role of Nrf2 in mitochondria dynamics/mitophagy is currently limited.

## 3. Mitochondrial Dynamics and Mitophagy

### 3.1. Mitochondrial Fission

**Figure 2:**
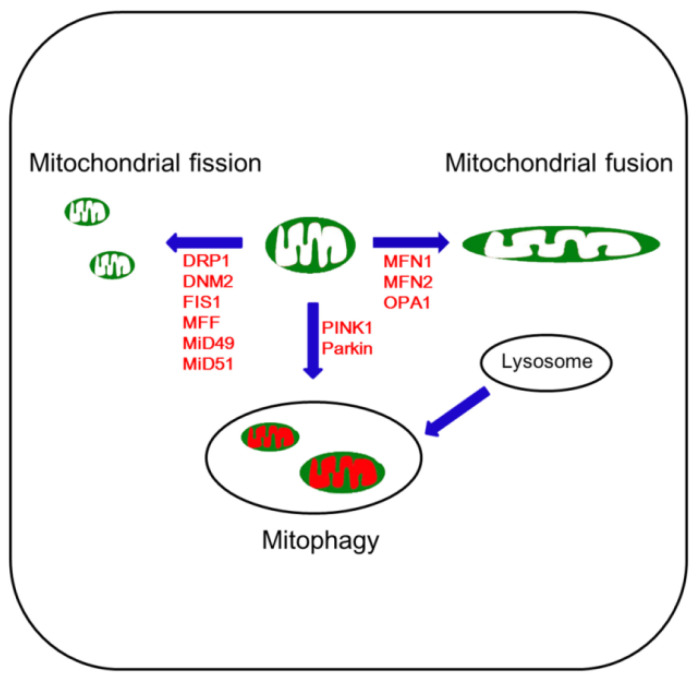
Schematic depiction of mitochondrial dynamics and mitophagy under physiological condition. Abbreviations: DMN2, dynamin-2; DRP1, dynamin-related protein-1; FIS1, fission protein 1 protein; MFF, mitochondrial fission factor; MFN1, mitofusin 1; MFN2, mitofusin 2; Mid49, mitochondrial dynamic proteins of 49; MiD51, mitochondrial dynamic proteins of 51; OPA1, optic atrophy 1; PINK1, phosphatase and tensin homolog (PTEN)-induced kinase 1.

Mitochondrial fission is required for mitochondrial motility and mitochondrial DNA (mtDNA) inheritance in the G2/M phase of the cell cycle. In addition, mitochondrial fission plays an important role in the regulation of mitochondrial size/shape and in the distribution of mitochondria throughout the cell body, especially in neurons. Mitochondrial fission creates new (daughter) mitochondria and segregates damaged mitochondria. It is also needed for mitophagy (see below) that is an essential autophagosomal process removing excess or damaged mitochondria to maintain mitochondria quality control and homeostasis [5,38]. However, fragmented mitochondria have a low yield of ATP synthesis, which in turn impairs the detoxification of excess ROS and extrusion of intracellular Ca^2+^ [23,24,39,40]. Thus, excessive mitochondrial fragmentation results in apoptosis under stress conditions through impaired bioenergetics, ROS generation, loss of MMP, dysfunction of endogenous respiration and release of pro-apoptotic factors from mitochondria [1,40,41,42,43,44,45,46,47]. In mammals, mitochondrial constriction and scission are carried out by dynamin-related protein-1 (DRP1, also known as dynamin-1 (DNM1)), DNM2, fission protein 1 protein (FIS1), mitochondrial fission factor (MFF), and the mitochondrial dynamic proteins of 49 (MiD49) and 51 kDa (MiD51) [17,48,49,50] (Figure 2).

The large guanosine 5′-triphosphate (GTP)-ase DRP1 plays a crucial role in mitochondrial fission. DRP1 is a cytosolic protein that is dynamically recruited to mitochondrial membranes where it oligomerizes and drives membrane constriction in a GTP-dependent manner. During mitochondrial division, DRP1 is recruited to the outer mitochondrial membrane (OMM) where it forms a ring-like structure around mitochondria leading to a narrowing of the membrane. In turn, GTP hydrolysis enhances this membrane constriction that marks a potential future site of mitochondrial scission. In addition, mitochondria-bound DRP1 puncta merge into a mature-sized DRP1 complex capable of moving laterally along the mitochondrial tubule, thereby inducing constriction and eventually fission [17,48,49,50]. DRP1 activity is regulated by post-translational modifications. During mitosis, DRP1 is phosphorylated on serine (S) 616 by cyclin-dependent kinase 1/cyclin B kinase, protein kinase C (PKC), Ca^2+^/calmodulin-dependent kinase II (CaMKII) or extracellular-signal-regulated kinase 1/2 (ERK1/2). This phosphorylation stimulates its oligomerization and induces mitochondrial fission. In contrast, protein kinase A (PKA) phosphorylates DRP1 on S637, which detaches DRP1 from mitochondria and subsequently inhibits fission. Thus, DRP1-S637 phosphorylation protects mitochondria from autophagosomal degradation during nutrient deprivation. Dephosphorylation of this site is mediated by calcineurin and PGAM family member 5 (PGAM5) [17,48,49,50]. FIS1, MFF, MiD49 and MiD51 are integral membrane proteins of the OMM, which act as receptors that recruit DRP1 to the mitochondrial surface. FIS1 and MFF recruit DRP1 to the OMM. Both FIS1 and MFF have C-terminal single transmembrane domains that anchor them to the outer mitochondrial membrane. FIS1 is a limiting factor in DRP1-mediated fission, but MFF-dependent fission does not require FIS1 [17,48,49,50]. MiD49 and MiD51 are also anchored to the OMM through N-terminal transmembrane domains. MiD49 and MiD51 mediate DRP1 recruitment in the absence of FIS1 and MFF [51]. In contrast to DRP1, DNM2 only localizes transiently on the OMM and appears in only one of daughter mitochondria following scission [52].

### 3.2. Mitochondrial Fusion

Mitochondrial fusion maintains the integrity of mitochondria and compensates their functional defects by facilitating the spreading of molecules and mtDNA throughout the entire mitochondrial compartment. Therefore, mitochondrial fusion is critical to avoid accumulation of mitochondrial mutations. Impaired mitochondrial fusion results in a loss of MMP. However, excessive mitochondrial fusion abrogates the segregation and elimination of damaged mitochondria. Aberrant mitochondrial elongation also inhibits respiratory function in mitochondria, which triggers excessive ROS production and leads to the abolishment of mitochondrial transports in dendrites or axons, thereby subsequently inducing ATP deficiency in peripheral sites [47,53,54,55,56,57,58,59,60,61].

Mitochondrial fusion is regulated by mitofusins 1 and 2 (MFN1 and MFN2) and optic atrophy 1 (OPA1) (Figure 2) [53,62]. MFN1 and MFN2 are transmembrane proteins that bind and hydrolyze GTP to catalyze the unification of adjacent OMMs. MFN1 and MFN2 contain the N-terminal GTPase domain as well as coiled-coil motif-containing two heptad repeat regions (HR1 and HR2) and two C-terminal transmembrane domains. In mitochondrial fusion, MFN1 and MFN2 mediate tethering of apposing mitochondria prior to membrane fusion. Although MFN1-MFN1 tethering is more efficient than MFN2-MFN2 tethering, MFN1 and MFN2 can form heterotypic complexes. Furthermore, these MFN1-MFN2 heterotypic complexes are more efficient in fusion than homotypic complexes [53,62]. In contrast, OPA1 is anchored to the inner mitochondrial membrane (IMM) by the N-terminal transmembrane domain, which leaves the bulk of the molecule in the intermembrane space. Because of the two distinct membrane systems of mitochondria, completing mitochondrial fusion in one step is topologically impossible. MFN1/2 initiate the tethering and fuse the OMM of apposing mitochondria, which allows subsequent fusion through the function of OPA1 [53,62]. While OPA1 requires the MFN1 isoform for mitochondrial fusion, MFN1 and MFN 2 exert the OMM fusion first, and OPA1 then mediates the IMM fusion. These two phases are coordinated and occur almost simultaneously [53,62].

### 3.3. Mitophagy

Mitophagy is a process composed of the selective sequestration of excessive or damaged mitochondria by the autophagosome and subsequent lysosome-mediated degradation [5]. The phosphatase and tensin homolog (PTEN)-induced kinase 1 (PINK1) and the E3 ubiquitin ligase parkin are highly solicited during this process (Figure 2). Under stressful conditions, PINK1 selectively recognizes and accumulates on damaged mitochondria. Thereafter, PINK1 recruits parkin that catalyzes the degradative ubiquitination of various proteins including MFN1 and MFN2 on the surface of defective mitochondria, which in turn promotes lysosome-mediated mitochondrial degradation [37,63,64]. The autophagic adaptor protein sequestosome 1 (SQSTM1)/p62 is also involved in mitophagy. p62 is localized in mitochondria under physiological conditions. p62 deficiency leads to mitochondrial dysfunction with defective MMP, genome integrity and energy production. p62 is required for the mitochondrial import of TFAM, which maintains mitochondrial integrity. In addition, p62 enhances the mitochondrial import of transcription factors and increases mitochondrial energy production [5,63]. Under oxidative stress, p62 expression is up-regulated via Nrf2 signaling, which creates a positive feedback loop between Nrf2 and p62, because p62 and Nrf2 bind competitively to Keap1 protein. Under physiological conditions, mitophagy prevents accelerated cellular senescence and programmed cell death [5,63]. However, excessive mitophagy is detrimental to cellular homeostasis [37,63,64].

## 4. Nrf2, Mitochondrial Dynamics, and Mitophagy in Neurological Diseases

### 4.1. Alzheimer’s Disease

Alzheimer’s disease (AD) accounts for almost three-quarters of cases of dementia, characterized by progressive decline of memory and cognitive function as well as changes in behavior and personality, with severe brain neurodegeneration. The cardinal features of AD pathology are amyloid plaques and neurofibrillary tangles (NFTs), accompanied by neuropil threads, dystrophic neurites, reactive astrogliosis, microglial activation and cerebral amyloid angiopathy. Mixed pathology frequently occurs particularly in older individuals and includes vascular disease and Lewy bodies (LBs) [65]. Amyloid plaques are extracellular accumulations principally composed of abnormally folded amyloid β (Aβ) with 40 or 42 amino acids (Aβ40 and Aβ42, respectively), produced by amyloid precursor protein (APP) metabolism. Aβ deposition develops in the isocortex and subsequently in subcortical structures. A lesser extent of Aβ accumulation occurs in the entorhinal cortex and the hippocampus [65,66]. Aβ pathology reaches a plateau early in the symptomatic phase of the disease [65,67]. NFTs are primarily composed of paired helical filaments comprising hyperphosphorylated Tau. Unlike amyloid plaques, Tau pathology typically begins in the allocortex of the medial temporal lobe (entorhinal cortex and hippocampus) before spreading to the associative isocortex, whereas primary sensory, motor, and visual areas are relatively spared. The loss of neurons and synapses typically parallels NFTs formation, and as such the clinical features and severity of AD correlate best with NFT pathology [65,66]. Both Aβ and Tau decrease synapse strength by increasing the endocytosis and degradation of various proteins including α-amino-3-hydroxy-5-methyl-4-isoxazole-propionic acid (AMPA) receptors, N-methyl-D-aspartate (NMDA) receptors, postsynaptic density protein 95 (PSD-95), as well as by degrading mitochondrial dynamics/functions [68,69].

**Figure 3:**
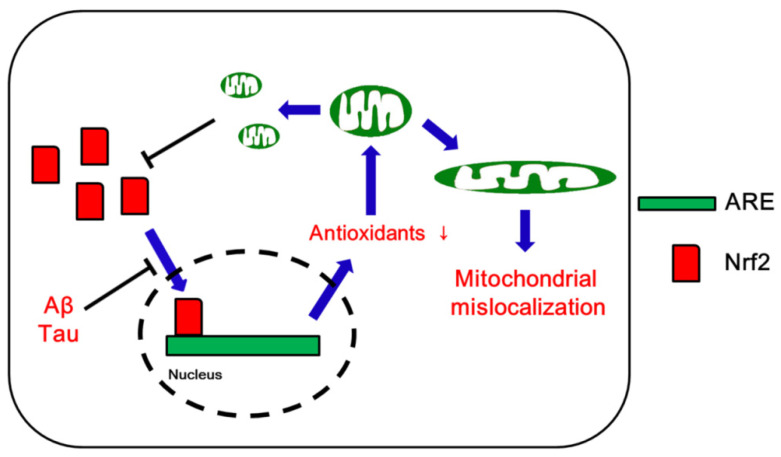
Schematic depiction of likely roles of Nrf2 in mitochondrial dynamics in Alzheimer’s disease (AD).

Nrf2 protein is detected in both the nucleus and the cytoplasm of neurons in the normal human hippocampus, with predominance in the nucleus (Figure 1). In hippocampal neurons from AD brains, however, Nrf2 is predominantly localized in the cytoplasm rather than the nucleus, indicating impaired Nrf2-mediated transcription of antioxidant enzymes (Figure 3). Indeed, the expression levels of Nrf2-regulated antioxidant enzymes such as SOD1 and catalase are reduced in human AD brains. Furthermore, overexpression of Nrf2 enhances neuroprotection against Aβ toxicity. Therefore, it is likely that Nrf2 may not be responding properly to oxidative stress in AD brains, contributing to neuronal dysfunction and/or loss [70].

Mitochondria are one of the prime targets for APP. APP affects mitochondrial import channels. Aβ also interacts with numerous mitochondrial proteins and leads to mitochondrial dysfunction. Aβ perturbs mitochondrial Ca^2+^ homeostasis via decreased Ca^2+^ ATPase activity and MMP, resulting in the formation of mitochondrial permeability transition pores in the inner mitochondrial membrane and thereby leading to ATP depletion, cytochrome c release and ROS production. Tau also binds to mitochondria and causes mitochondrial dysfunction and impaired energy metabolism. Aβ and Tau reduce mitochondrial protein levels mainly related to complexes I and IV of the electron transport chain, MMP, and ATP synthesis. Furthermore, Aβ and Tau synergistically impair oxidative phosphorylation, where dysregulation of complex I is Tau-dependent and dysregulation of complex IV is Aβ-dependent. Indeed, activities of cytochrome oxidase and mitochondrial ATP synthase are decreased in AD brains [37,71].

In addition, the pathogenesis of AD is linked to defective mitochondrial dynamics, impaired mitochondrial movement and altered mitophagy. In AD neurons, synaptic mitochondria are more susceptible to Aβ-induced mitochondrial dysfunction as compared to non-synaptic mitochondria. During the aging process, Aβ accumulation, mitochondrial dysfunction, increased mitochondrial permeability transition, decreased mitochondrial respiration and cytochrome c oxidase activity are all facilitated in synaptic mitochondria. Furthermore, APP and Aβ lead to alterations in mitochondrial morphology and distribution, and impair modulation of the mitochondrial fusion/fission machinery. Aβ causes decreased mitochondrial numbers, mitochondrial velocity and mitochondrial length, which affect mitochondrial anterograde and retrograde axonal transport in neurons. Interestingly, in AD brains Aβ co-localizes with DRP1 and facilitates mitochondrial fragmentation in vulnerable neurons [37,71]. In addition, an increase in autophagic vesicles containing mitochondria is found in pyramidal neurons from AD patients, suggesting enhanced mitophagy [72,73]. In contrast, brain tissues obtained from AD patients also show a mitochondrial fission arrest phenotype resulting in elongated interconnected organelles (mitochondria-on-a-string, MOAS) at the final stages of the mitochondrial fission process, due to reduced GTPase activity. MOAS accumulation indicates inhibition of axonal trafficking of mitochondria. MOAS formation allows mitochondria to sustain cell viability during energy deprivation, which plays a role in neuronal survival. MOAS formation also inhibits mitophagy, thereby preserving a low level of mitochondrial function under extreme stress [74]. In addition, Aβ abrogates mitochondrial anterograde and retrograde axonal transports in neurons. Indeed, the decreased DRP1 level evokes the abnormal mitochondrial distribution and the presence of elongated mitochondria in fibroblasts from sporadic AD patients [37,71]. Similarly, Tau promotes neurodegeneration via aberrant mitochondrial elongation [56]. However, little is known about the involvement of Nrf2 in mitochondrial dynamics/mitophagy in AD.

In primary mouse hippocampal neurons (HT22 cells), mutant APP (mAPP) increases levels of mRNA and protein levels of mitochondrial fission genes (DRP1 and FIS1), while it decreases levels of proteins involved in mitochondrial fusion (MFN1, MFN2 and OPA1), biogenesis (PGC1α, nuclear respiratory factor 1 (NRF1), Nrf2 and TFAM) and mitophagy (PINK1). DRP1 activity is also increased in mAPP-HT22 cells. The number of mitochondria is increased, but mitochondrial length is reduced in mAPP-HT22 cells. These findings suggest that accumulation of mAPP and Aβ may be responsible for abnormal mitochondrial dynamics and defective biogenesis, which would ultimately induce excessive mitochondrial fragmentation causing mitochondrial and synaptic damage in AD neurons. Since mAPP decreases the expression levels of Nrf2 and PINK1, the reduced antioxidant defense and defective mitophagy may be also involved in the mitochondrial defects in AD brains [75].

Similarly, the expression levels of MFN1, MFN2, OPA1 and NRF2 are reduced, but those of DRP1 and FIS1 are increased in mAPP neuroblastoma cells (N2a cells), accompanied by reduced protein levels of mitochondrial biogenesis factors (PGC1α, NRF1, Nrf2, and TFAM). Treatment of microRNA (miR)-455-3p against abnormal APP treatment results in an increase in mitochondrial length and a decrease in mitochondrial number. Furthermore, it suppresses fission proteins (DRP1 and FIS1) and increases fusion proteins (MFN1, MFN2, and OPA1). miR-455-3p also increases the protein levels of mitochondrial biogenesis. miR-455-3p decreases mitochondria number, but increases mitochondrial length [76]. Therefore, accumulation of mAPP results in abnormal mitochondrial fission and defective Nrf2-mediated antioxidant system, accompanied by reduced mitophagy and ultimately leading to neuronal degeneration.

APP over-expressing mice (APP mice) show increased levels of mitochondrial fission proteins (DRP1 and FIS1) and decreased levels of fusion (MFN1, MFN2 and OPA1), biogenesis (PGC1α, NRF1, Nrf2 and TFAM) and mitophagy proteins (PINK1), as compared to age-matched non-transgenic WT mice [77]. Tau over-expressing mice also show increased DRP1 and FIS1 levels but decreased MFN1, MFN2, OPA1, PINK1, and Nrf2 levels, as compared to WT mice [62]. Thus, it is likely that APP and Tau may facilitate mitochondrial fission, accompanied by reduced Nrf2 activity, in AD brains.

Interestingly, double mutant mice with APP or Tau over-expression and partial DRP1 deletion (*APP×Drp1^+/−^* and *Tau×Drp1^+/−^* mice, respectively) show decreased FIS1 expression levels but increased MFN1, MFN2, OPA1, and Nrf2 levels, as compared to APP- and Tau over-expressing mice, respectively [78,79]. *Tau×Drp1^+/−^* mice show increase in mitochondrial function concomitant with reduced Tau phosphorylation [78]. Similarly, *APP×Drp1^+/−^* mice demonstrate enhanced mitochondrial function in AD neurons with reduced Aβ levels [79]. Partial DRP1 deletion also elevates mitochondrial biogenesis, indicating that reduced DRP1 activity is protective against mAPP- and Tau-induced mitochondrial toxicities. Therefore, it is postulated that Aβ-DRP1 and/or Tau-DRP1 complex would cause mitochondrial dysfunction and defective axonal/dendritic mitochondrial transport, leading to synaptic damage via insufficient ATP synthesis in AD neurons [80,81]. Furthermore, since partial DRP1 deletion decreases Tau toxicity, Aβ production and mitochondrial dysfunction, it is also likely that excessive DRP1-mediated mitochondrial fission may decrease Nrf2 activity in the AD brain (Figure 3 and Table 1).

### 4.2. Parkinson’s Diseases

**Figure 4:**
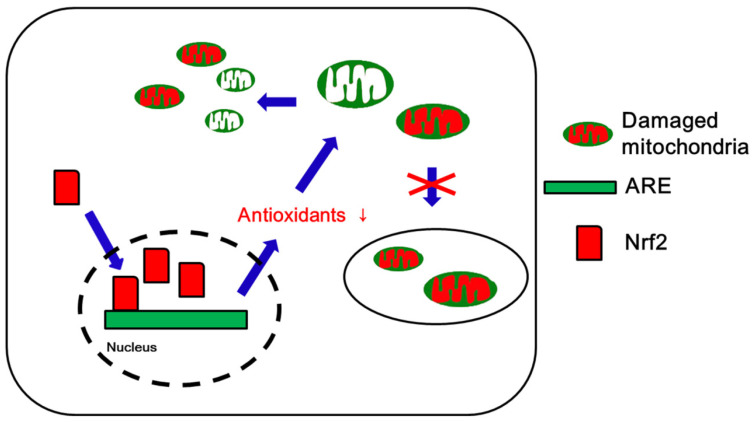
Schematic depiction of likely roles of Nrf2 in mitochondrial dynamics in Parkinson’s disease (PD).

Parkinson’s disease (PD) is a neurodegenerative disease characterized by motor impairment including resting tremor, bradykinesia, rigidity and postural instability, as well as showing non-motor symptoms such as sleep perturbations, constipation, cognitive impairment or depression. The degeneration of dopaminergic neurons in the substantia nigra is a main event in the pathogenesis of PD [80]. The diagnostic hallmark of PD in both familial and sporadic cases is the presence of LBs as abnormal protein aggregates developing inside neurons. The main constituent of LBs is α-synuclein, a small protein with 140 amino acids. α-Synuclein is abundant in presynaptic nerve terminals, and regulates synaptic transmission and dopamine levels adjustment. Increased ROS production plays an important role in α-synuclein proteostasis, which is counteracted by Nrf2 activity. Nrf2 decreases α-synuclein aggregates and prevents the loss of dopaminergic neurons. Indeed, Nrf2 deficiency enhances dopaminergic neuron loss, neuroinflammation and protein aggregation [70,80]. Unlike what is observed in AD, Nrf2 shows strong nuclear localization in PD nigral neurons (Figure 4). However, this nuclear localization of Nrf2 is insufficient to protect neurons from degeneration [70].

Similar to AD, dysfunction in mitochondrial integrity is also associated with PD [37,81]. A deficiency in mitochondrial respiratory electron transport chain NADH dehydrogenase (complex I) activity and a reduced level of mitochondrial α-alpha-ketoglutarate dehydrogenase (the rate limiting enzyme of TCA cycle) are observed in the substantia nigra of PD patients [37,81,82,83]. Thus, defective mitochondrial bioenergetics and reduced mitochondrial complex activity are involved in PD pathogenesis. Furthermore, a number of studies have suggested that mtDNA mutations and polymorphisms related to mitochondrial function and/or oxidative stress responses might play an important role in PD pathogenesis [37,81,82,83]. Genetic variations in NADH dehydrogenase ubiquinone flavoprotein 2 (encoding a subunit of mitochondrial complex I) are possibly associated with idiopathic PD. Heteroplasmic mutations in *NADH:ubiquinone oxidoreductase ND5* (a mitochondrial gene encoding a complex I subunit) are also reported in PD brain [37,81,82,83]. Furthermore, increased levels of mtDNA deletions/rearrangements are associated with neurodegeneration in PD. These mtDNA deletions are also relevant for respiratory chain deficiency in PD patients. A G11778A mtDNA point mutation in a subunit of mitochondrial complex I has been reported in a family with PD and multisystem degeneration. Mutations in mortalin (a mitochondrial chaperone regulating mitochondrial biogenesis and mitochondrial homeostasis) are also observed in PD patients [37,81,82,83].

On the other hand, the mitochondrial import and accumulation of α-synuclein increases ROS generation and impairs mitochondrial complex I in the substantia nigra and striatum of PD brain. α-Synuclein localization on mitochondrial membranes increases the release of cytochrome c and the oxidative modification of mitochondrial components. Thus, α-synuclein enhances mitochondrial fragmentation [37,81,82,83]. In particular, *PINK1* mutations are responsible for an autosomal recessive familial form of early-onset PD. PINK1 is detected in LBs within the brains of sporadic PD patients. Polymorphisms in the mitochondrial translation initiation factor 3 (MTIF3, a protein interacting with PINK1) are also involved in the pathogenesis of PD. Mutations in *PINK1* provoke deficits in mitochondrial respiration and ATP synthesis, and increase α-synuclein aggregation. Furthermore, *PINK1* mutants perturb the opening of the mitochondrial permeability transition pore, MMP, cytochrome c release and Ca^2+^ homeostasis [37,81,82,83]. PINK1 also binds to TNF receptor-associated protein 1 (TRAP1, a mitochondrial molecular chaperone) in mitochondria. PINK1 phosphorylates TRAP1 and protects cells against oxidative stress by suppressing cytochrome c release from mitochondria, which is impaired by PD-associated mutations in *PINK1* [37,81,82,83]. *Parkin* mutations are involved in early onset autosomal recessive juvenile PD. Heterozygous deletions and single-nucleotide polymorphisms of the *Parkin* gene are involved in early- and late-onset idiopathic PD patients, respectively. Because parkin promotes mitophagy and mitochondrial clearance by catalyzing mitochondrial ubiquitination, mutations in *parkin* increase mitochondrial aggregation [37,81,82,83]. Mutations in *DJ-1* are also relevant in autosomal recessive early-onset PD, since levels of DJ-1 and of the disassembled DJ-1 high molecular weight complex are decreased in the mitochondria obtained from PD brains [37,81,82,83].

In addition, dysregulation of mitochondrial dynamics is linked to the pathogenesis of PD. PINK1 regulates mitochondrial dynamics through DRP1 and FIS1. *DJ-1* mutation causes impairment of DRP1-mediated mitochondrial dynamics. PINK1 and parkin regulate axonal transport of mitochondria. Furthermore, PINK1 and parkin ubiquitinate MFN1 and MFN2 for selective removal of damaged mitochondria. Thus, mutations in *PINK1* and *parkin* interrupt mitochondrial dynamics and mitophagy in PD [37,81,82,83]. Although Ammal Kaidery et al. [84] have recently reviewed the crosstalk between the Nrf2 system and mitochondrial function, the relationship between Nrf2 and mitochondrial dynamics/mitophagy in patients has not been sufficiently eludidated.

In the 6-hydroxydopamine (6-OHDA)-induced rat model of PD, the cytosolic and nuclear protein levels of Nrf2 are markedly reduced, which is accompanied by facilitated DRP1-mediated mitochondrial fission [85]. In the 1-methyl-4-phenyl-1,2,3,6-tetrahydropyridine (MPTP) model of PD, the expression levels of FIS1 are elevated concomitantly with the reduced levels of MFN2 and Nrf2 [86], although Mendes et al. [87] have reported that MPTP transiently increases the expression of Nrf2 and parkin. Thus, it is likely that reduced Nrf2 expression/activity may be relevant for mitochondrial fragmentation and impaired mitophagy in PD (Figure 4 and Table 2).

### 4.3. Nrf2, Mitochondrial Dynamics, and Mitophagy in Huntington’s Disease

Huntington’s disease (HD) is an autosomal dominant and fatal neurological disorder caused by the loss of GABAergic inhibitory spiny projection neurons in the striatum due to the abnormal triplet expansion of a CAG repeat in exon-1 of the gene responsible for HD that results in elongated polyglutamine stretches in the protein product known as mutant huntingtin (Htt) [88,89,90,91]. HD is characterized by involuntary movements, chorea, dystonia, changes in personality and cognitive decline, associated with selective degeneration of medium-sized spiny striatal projection neurons and depletion of their neurochemical components. Htt aggregation directly enhances ROS generation that promotes cell toxicity [88,89,90,91]. Mutant Htt protein also impairs the function of PGC-1α and leads to abnormalities in mitochondrial function and energy metabolism. Impaired PGC-1α transcription and activity result in down-regulation of ROS defense genes encoding SOD1, SOD2, and GPx, suggesting that both mitochondrial dysfunction and oxidative damage may contribute to the pathogenesis of HD [88,89,90,91]. Although several *in vitro* studies show a protective effect of Nrf2 activation against Htt-induced toxicity, the activation of the Nrf2 system in striatal cells in response to ROS is disrupted in HD patients due to the concurrent activation of the autophagy pathway [37,88,89,90,91].

**Figure 5:**
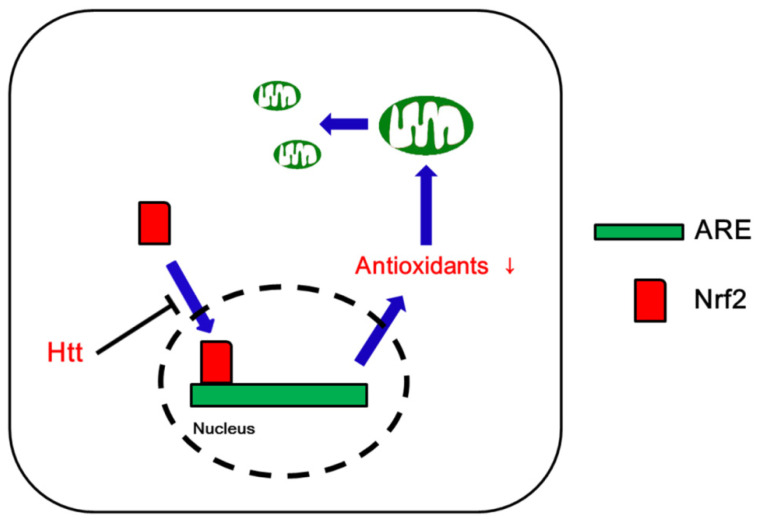
Schematic depiction of likely roles of Nrf2 in mitochondrial dynamics in Huntington’s disease (HD).

HD patients show disturbances in mitochondrial bioenergetics, such as reductions in activities of oxidative phosphorylation components as well as the TCA cycle, mitochondrial complexes II-IV and aconitase, without alterations in complex I activity [37,88,89,90,91]. In addition, variations in mitochondrial haplogroup H are related to altered ATP levels, mitochondrial dysfunction and age of onset in HD. Mutant Htt directly binds to the OMM and increases the susceptibility of mitochondria to the Ca^2+^-induced permeability transition and cytochrome c release, which induce oxidative stress. Mutant Htt also impairs mitochondrial motility and mitochondrial functions. Furthermore, mutant Htt binds to DRP1 and increases its mitochondrial fission enzymatic activity [37,88,89,90,91]. Indeed, DRP1 and FIS1 levels are higher, whereas MFN1, MFN2, and OPA1 levels are lower in brain specimens from HD patients, as compared to controls. These findings indicate that high fission protein levels are responsible for mitochondrial fragmentation and mitochondrial swelling in HD neurons [92,93,94]. Although the profiles of Nrf2 in HD patients are still unknown, striatal cells expressing mutant Htt with 111 glutamines (STHdh Q111/Q111) cells show reduced Nrf2 activity, accompanied by the increased expression of DRP1 and decreased expression of OPA1 [95,96,97] (Figure 5 and Table 3).

In addition, hyperactivation of autophagy signaling partially contributes to the impairment of Nrf2 activation by oxidative stress in STHdhQ111/Q111 cells. Mutant Htt-induced interference with CREB-binding protein (CBP)/p300 is also postulated as a crucial factor leading to the abrogation of Nrf2 signaling in STHdhQ111/Q111 cells. Therefore, mutant Htt may disrupt Nrf2 signaling leading to impaired mitochondrial dynamics that enhance susceptibility to oxidative stress [96].

### 4.4. Nrf2, Mitochondrial Dynamics and Mitophagy in Cerebrovascular Disease

Stroke is defined by sudden interruption of the local blood supply and by initiation of an anoxic and hypoglycemic state in the affected brain tissue. Under this condition, neuronal depolarization leads to the release of the neurotransmitter glutamate, which is the activator of the ionotropic NMDA receptor, resulting in Ca^2+^ overload and excessive ROS production by mitochondria [98,99,100,101,102]. Furthermore, degradation of structural proteins of the vascular wall and loss of brain–blood barrier (BBB) integrity further enhance tissue oxygenation, which exacerbates ROS production. Although there are few studies on the alterations of the Nrf2 system in human patient specimens, Nrf2 pathway activators up-regulate the expression of tight junctional proteins (TJ) and promote redox metabolic functions and ATP synthesis with mitochondrial biogenesis, which protect neurons from stroke in animal models [98,99,100,101,102,103,104].

Numerous animal studies demonstrate alterations in mitochondrial dynamics/mitophagy in models of ischemic stroke, although data obtained from patient specimens are limited. The impairments of mitochondrial respiratory function and MMP activate a cascade of events that leads to neuronal death after ischemia. Depolarized mitochondria initiate excessive ROS production, decreased ATP generation and PINK1 accumulation. In stroke, abnormalities in MMP and ROS over-production induce neuronal injury [100,101,102,103,104,105]. DRP1-mediated mitochondrial fission is an early event required for ischemic neuronal death, which is associated with the failure of bioenergetics and the increased ROS production. In the ischemic core, the expression levels of DRP1 and OPA1 progressively decrease until 2 days after the ischemic insult. In the ischemic penumbra, the protein levels of both DRP1 and OPA1 increase at 2 days after ischemia, and recover to the basal level 7 days after the insult. Ischemic insults reduce MFN2 expression, leading to mitochondrial dysfunction and disruption of Ca^2+^ homeostasis [103,104]. Interestingly, neurons invulnerable to ischemia can shift their mitochondrial dynamics toward fusion after extensive fission: CA1 hippocampal neurons (a cell population vulnerable to ischemia) show mitochondrial fragmentation as early as 2 h after insults. Mitochondrial fragmentation is also detected in CA3 neurons and dentate granule cells (cell populations invulnerable to ischemia), but fragmented mitochondria are re-fused in these neurons 24 h after the insult [103,105]. However, the role of mitophagy in the development of ischemic brain injury remains controversial. Most studies demonstrate that mitophagy protects neurons from cell death signaling cascades, while excessive mitophagy induction leads to neuronal death in neonatal stroke [65,103,106]. Furthermore, the types of cerebrovascular insults distinctly affect Nrf2 activity and mitochondrial dynamics/mitophagy: ischemic stroke results in elevated DRP1-mediated mitochondrial fragmentation and downregulation of Nrf2 activity. However, subarachnoid hemorrhage (SAH) increases Nrf2 expression levels but decreases OPA1 expression levels [107,108,109] (Table 4). Therefore, further studies are needed to elucidate the relationship between Nrf2 and mitochondrial dynamics/mitophagy in cerebrovascular diseases.

### 4.5. Nrf2, Mitochondrial Dynamics and Mitophagy in Epilepsy

Epilepsy is a complex and chronic neurological disorder defined by the occurrence of unprovoked seizures [110,111]. Unlike the aforementioned neurological diseases, a significant increase in *Nrf2* mRNA expression is observed in hippocampal tissue from patients with epilepsy. Similarly, pilocarpine-induced status epilepticus (SE) strongly induces *Nrf2* mRNA and its target genes (heme oxygenase-1 (HO-1), NQO1, and GSTs) in mice. Furthermore, injection of adeno-associated virus (AAV) vector coding for human *Nrf2* into the hippocampus of epileptic mice attenuates the number and duration of generalized seizures. Thus, *Nrf2* mRNA up-regulation may be an adaptive response to repeated seizure activity [112]. However, Liu et al. [113] have reported that *Nrf2* gene variations increase temporal lobe epilepsy (TLE, a prevalent form of epilepsy) and drug-resistant epilepsy (DRE), while *Keap1* gene variations play a protective role in DRE. Since Nrf2 modulates the expression of a large number of genes encoding detoxification enzymes, antioxidant proteins, xenobiotic transporters and other stress response proteins involved in many cellular and molecular pathways, the role of Nrf2-Keap1 signal in ictogenesis (the processes of transition to a seizure) in epilepsy has been elusive.

Patients with mitochondrial mutations often present with epilepsy as a phenotypic manifestation of the disease. A ketogenic (a high-fat) diet up-regulates the neuronal expression of genes involved in TCA cycle, oxidative phosphorylation and glycolysis, along with improving mitochondria complex activities and boosting mitochondrial biogenesis in epilepsy patients and animal models. In particular, a ketogenic diet is effective in patients suffering from seizures due to glucose transporter 1 (GLUT-1) deficiency and pyruvate dehydrogenase complex deficiency [114]. Similar to other neurological diseases, seizure activity results in excessive Ca^2+^ accumulation that provokes mitochondrial swelling, permeability transition pore opening, activation of mitochondrial proteins, release of cytochrome c, activation of caspases and loss of OMM integrity [110,111,112,114]. Abnormal mitochondrial distribution, altered mitochondrial motility, decreased MMP and diminished mitochondrial respiration are also relevant for the pathogenesis of epilepsy [114,115]. Interestingly, a *de novo* heterozygous *DRP1* pathogenic variant was identified in a female neonate with lethal encephalopathy characterized by cerebral dysgenesis, seizures, lactic acidosis, the elevated very long chain fatty acids and abnormal mitochondrial and peroxisomal elongation [17]. Vanstone et al. [116] have also reported that fibroblasts isolated from patients who presented with developmental delay and refractory epilepsy show hyperfusion of the mitochondrial network due to DRP1-mediated mitochondrial fission defect, despite no differences in bioenergetics.

Consistent with these previous studies, we have reported that aberrant mitochondrial elongation induced by impaired DRP1-mediated mitochondrial fission leads to programmed necrotic neuronal death in CA1 pyramidal cells without altering the expression levels of MFN1/2 and OPA1 in the pilocarpine-induced rat model of SE [47,58,117]. Furthermore, dysfunction of mitochondrial fission facilitates the degeneration of dentate granule cell that is one of the resistant neuronal populations to various harmful stresses [59]. However, DRP1 activation induces mitochondrial fragmentation and apoptosis in parvalbumin interneurons that play an important role in the adaptation to repetitive spikes [46]. Recently, we have also reported that 2-cyano-3,12-dioxo-oleana-1,9(11)-dien-28-oic acid methyl ester (CDDO-Me; RTA 402; a potent Nrf2 activator) induces extracellular-signal-regulated kinase 1/2 (ERK1/2)-mediated DRP1 activation, and attenuates SE-induced CA1 neuronal death by facilitating mitochondrial fission. However, CDDO-Me does not prevent PV cell loss, even though it abolishes 4-hydroxy-2-nonenal (4-HNE, the end-product of lipid peroxidation) signals in both cell populations. These findings indicate that CDDO-Me may differentially affect regional specific neuronal death patterns induced by SE, independent of ROS generation [47]. Regardless of the limited availability of clinical data, these findings indicate that the roles of Nrf2 and mitochondrial dynamics/mitophagy in epilepsy may be distinct from other neurological diseases. Future studies are needed to elucidate the status of the Nrf2 system and mitochondrial dynamics/mitophagy in patients with epilepsy.

## 5. Concluding Remarks and Future Perspectives

**Figure 6:**
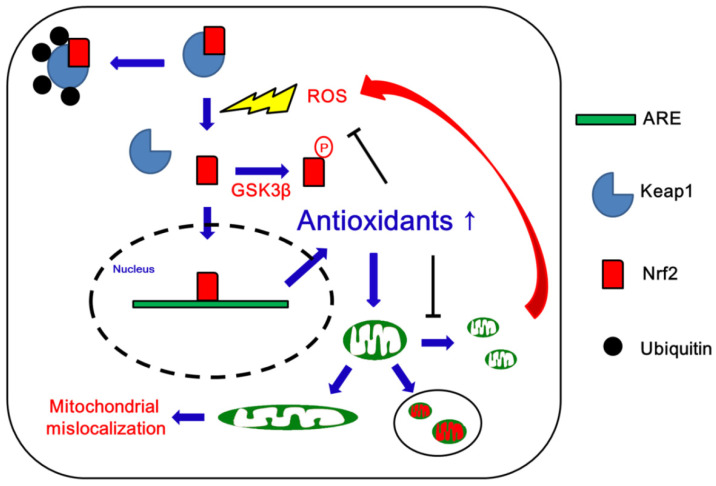
Schematic depiction of Nrf2 and mitochondrial dynamics under physiological conditions. Oxidative stress activates Nrf2-mediated antioxidant defense mechanisms that prevent excessive mitochondrial fission and inhibit further ROS synthesis by mitochondria. In addition, Nrf2 activation facilitates mitophagy. In general, impairments of the Nrf2 system and mitochondrial dynamics/mitophagy may be involved in the pathogenesis of various neurological diseases, even though the precise profiles of Nrf2 and mitochondrial dynamics/mitophagy are distinct in each disease type. However, aberrant mitochondrial elongation may also induce mitochondrial mislocalization leading to neuronal damage in AD and epilepsy.

The present article summarizes recent reports regarding the relevance of the Nrf2 system for mitochondrial dynamics/mitophagy in various neurological diseases. In AD, excessive DRP1-mediated mitochondrial fission may cause defective Nrf2-mediated antioxidant response, accompanied by reduced mitophagy. An arrest of mitochondrial fission is also involved in the pathogenesis of AD. In PD and HD, disruption of Nrf2 signaling leads to impaired mitochondrial dynamics that enhance susceptibility to oxidative stress. Stroke results in elevated mitochondrial fragmentation and downregulation of Nrf2 activity, while SAH increases Nrf2 expression accompanied by impaired mitochondrial fusion. In epilepsy, *Nrf2* mRNA expression is up-regulated concomitantly with hyperfusion of the mitochondrial network due to defective DRP1-mediated mitochondrial fission. As outlined above, a general pattern that emerges is that the down-regulation of Nrf2 expression may be highly relevant for mitochondrial fragmentation in neurological diseases, even though the precise profiles of Nrf2 and mitochondrial dynamics/mitophagy are distinct in each disease type (Figure 6).

Based on these considerations, the main questions that remain to be elucidated in future studies are the following: (1) Is there a common cause provoking Nrf2 pathway dysfunction and/or mitochondrial dynamics/mitophagy impairment in neurological diseases, and, if so, what is it? (2) What are the sources of exogenous and/or endogenous ROS involved in these processes? (3) Can altered energy production and ROS production induced by abnormalities of mitochondrial dynamics/mitophagy directly activate or repress Nrf2? (4) Can other antioxidant systems, such as glutathione, participate in the crosstalk between Nrf2 and mitochondrial dynamics/mitophagy? From a therapeutic perspective, the development of multi-target-directed agents acting on both the Nrf2 system and on mitochondrial dynamics/mitophagy would be highly desirable: For instance, Nrf2- or Nrf2 activator-based multi-targeted agents affecting mitochondrial dynamics/functions might improve outcomes in various neurological diseases. Therefore, further investigations concerning these questions and the development of multi-targeted agents for Nrf2 and mitochondrial dynamics/mitophagy will help to both understand the underlying pathological mechanisms of neurological diseases and assess ideal therapeutic targets in future studies.

## Figures and Tables

**Table 1:** Nrf2, mitochondrial dynamics and mitophagy in AD models (↑and↓ indicate increase and decrease, respectively.)

Model	Nrf2	Mitochondrial Responses	DRP1	FIS1	MFF	MFN1	MFN2	OPA1	PINK1	Ref.
APP HT22 cells	↓	Fission ↑	↑	↑		↓	↓	↓	↓	[75]
APP N2a cells	↓	Fission ↑	↑	↑		↓	↓	↓		[76]
Tau mice (vs. WT mice)	↓	Fission ↑	↑	↑		↓	↓	↓		[78]
*Tau×Drp1^+/−^*mice (vs. Tau mice)	↑	Fusion ↑	↓	↓		↑	↑	↑		[78]
APP mice (vs. WT mice)	↓	Fission ↑	↑	↑		↓	↓	↓	↓	[77,79]
*APP×Drp1^+/−^* mice (vs. APP mice)	↑	Fusion ↑	↓	↓		↑	↑	↑		[79]

**Table 2:** Nrf2, mitochondrial dynamics and mitophagy in PD models (↑and↓ indicate increase and decrease, respectively.)

Model	Nrf2	Mitochondrial Responses	DRP1	FIS1	MFF	MFN1	MFN2	OPA1	PINK1	Parkin	Ref.
6-OHDA (rat)	↓	Fission ↑	↑								[85]
MPTP (mouse)	↓	Fission ↑		↑			↓				[86]
MPTP (mouse)	↑ (transient)	-								↑ (transient)	[87]

**Table 3:** Nrf2, mitochondrial dynamics and mitophagy in HD models (↑and↓ indicate increase and decrease, respectively.)

Model	Nrf2	Mitochondrial Responses	DRP1	FIS1	MFF	MFN1	MFN2	OPA1	PINK1	Parkin	Ref.
STHdh Q111/Q111 cells	↓	Fission ↑	↑					↓			[95,96,97]

**Table 4:** Nrf2, mitochondrial dynamics and mitophagy in cerebrovascular disease models (↑ and ↓ indicate increase and decrease, respectively.)

Model	Nrf2	Mitochondrial Responses	DRP1	FIS1	MFF	MFN1	MFN2	OPA1	PINK1	Parkin	Ref.
Ischemia (mouse)	↓	Fission ↑	↑								[107]
SAH (rat)	↑	Fusion ↓						↓			[108,109]
